# Family and Work Lives of Lesbians in China: Implications for the Adult Worker Model

**DOI:** 10.3390/ijerph19116390

**Published:** 2022-05-24

**Authors:** Iris Po-Yee Lo, Emma H. Liu, Sam Wai-Kam Yu

**Affiliations:** 1 Department of Applied Social Sciences, The Hong Kong Polytechnic University, Hong Kong, China; 2 Department of Social Work, Hong Kong Baptist University, Hong Kong, China; emmahsliu@gmail.com (E.H.L.); samyu@associate.hkbu.edu.hk (S.W.-K.Y.)

**Keywords:** adult worker model, male breadwinner model, labour market, assisted reproduction, parent, gender, sexuality, heteronormativity, lesbian, work-family policy

## Abstract

This article examines the ways in which lesbians explore opportunities and navigate constraints in their family and work lives in urban China. It not only reveals Chinese lesbians’ difficulties in gaining equal access to the labour market and developing their desired family lives, but also discusses possible ways of enhancing the applicability of the adult worker model for sexual minority women. Previous research has indicated a shift from the male breadwinner model to the adult worker model, suggesting that both men and women are expected to join the labour market, and that women should not carry all the care responsibilities within the family. However, the model largely overlooks the interplay of gender and sexuality factors in shaping work and family lives. This article adopts a qualitative mixed-methods approach, including interviews with 20 Chinese lesbians and social media analysis, to examine lesbians’ experiences of taking part in the family as adults and in the wider economy as workers. It shows how gender norms, heteronormativity, and policy intersect in generating obstacles for Chinese lesbians to thrive as respectable adult workers. This has important implications for attempts to improve the adult worker model to fit better with people’s diverse work/family needs.

## 1. Introduction

A growing body of literature suggests that the male breadwinner model, in which men are expected to be the primary earners and women the primary carers, has been substantially eroded, as indicated by changes in family patterns and women’s increased participation in the labour market [1,2,3]. Such changes have been accompanied by a recognition of the need to explore alternatives to the male breadwinner model. One of the possible alternatives is the adult worker model [4,5]. This model builds on two assumptions. Firstly, all adults, regardless of gender, should engage in formal employment [6]. Secondly, the government should create favourable conditions for individuals, especially women, to join the labour market [4,5,6]. The government may achieve this by carrying out defamilisation measures, such as government provision of childcare services, with the aim of outsourcing care responsibilities from the family to formal sectors [5]. This implies that, in order to support the adult worker model, the government may assist people to organise not only their work lives but also their family lives.

This article builds a constructive bridge between discussions of the adult worker model and gender and sexuality studies. To date, there is still limited research on how to develop policy measures for building the adult worker model with reference to the experiences of sexual minority people. It is important to fill this gap for two reasons. Firstly, by discussing the applicability of this model to different groups, we can raise awareness of the difficulties they face in organising their work and family lives and the structural injustices they face. More importantly, it can inform the search for policy measures to address these difficulties, thereby identifying possible ways of improving the adult worker model.

This article focuses on lesbians in urban China. It not only reveals their difficulties in choosing ways of taking part (or not taking part) in the wider economy as a worker and in the family as an adult, but also provides fresh insights into the limitations of the applicability of the adult worker model and indicates potential ways of addressing these limitations. Lesbians are doubly disadvantaged by gender inequality and heterosexual norms in China, where women who are unmarried by their late 20s are stigmatised as ‘leftover women’ [7] and where same-sex marriage and parenthood are not legally recognised [8]. Moreover, Chinese lesbians have received much less academic or public attention than gay men [9,10]. Attending to the experiences of this oft-neglected yet socially marginalised group provides an important window into existing gendered, familial, and structural constraints, which remain unaddressed by policy, and their impact on individuals’ life chances in both the domestic and work spheres.

This article is organised into four parts. The first part provides background information about the adult worker model. It discusses why the model has received growing attention from policymakers and academics, and the extent to which it is applicable to problems confronting different social groups. The second part highlights the difficulties faced by lesbians when attempting to take part in the family as an adult and in the wider economy as a worker in China. We explain how the examination of these difficulties contributes to the discussion of the applicability of the adult worker model. The third part presents our data, which was collected through semi-structured interviews with 20 lesbian-identified women and a social media analysis of online articles related to lesbian issues in China. It provides empirical evidence of Chinese lesbians’ experiences and challenges in the workplace and the family. We conclude by discussing potential ways of creating favourable conditions for lesbians to reduce their difficulties in their work and family lives. We argue that such discussions can help to enhance the applicability of the adult worker model to sexual minority women.

### 1.1. Adult Worker Model

There has been growing concern over the effects of family policy on people’s experiences in the work and domestic spheres, and particularly on women’s opportunities to make choices when it comes to paid work and unpaid care work [11,12,13]. This could explain why the adult worker model has drawn increasing attention from policymakers and academic communities. Employment is widely seen as an important means of boosting economic growth and promoting social virtues, such as social inclusion and the development of human resources. This view is illustrated in two European Commission (EC) white papers on social and economic policy issued in 1994, the EU’s Lisbon Strategy agreed in 2000 and relaunched in 2005 [1], and other studies on social investment [14,15] and inclusive growth [16].

A number of family studies suggest that women may have more bargaining power over the allocation of unpaid care responsibilities if they engage in formal employment and secure a reasonable standard of living independently of their family relationships [11,12,13]. It is thus recommended that family care responsibilities should be outsourced to other sectors so that women can have more time and opportunities to develop their careers. For instance, government provision of childcare services may reduce the care responsibilities imposed on women, who are often expected to take up the traditional role of the family caregiver [5,6,17]. In short, the implementation of the adult worker model has the potential to bring positive welfare outcomes, such as encouraging more people to engage in the work economy, and assisting women, especially those who are able to earn a decent living, to gain more bargaining power over the allocation of unpaid care work in their family lives.

Despite the potential of the adult worker model, it is worth discussing whether it serves as a better alternative to the traditional male breadwinner model and whether everyone in need can access and benefit from the policy measures associated with the adult worker model. Notably, the male breadwinner model has two core defects. Firstly, it builds on the heterosexist belief that women are economically subordinate to men, who are expected to be the sole breadwinner within the heterosexual family. Women who stay at home without earning an independent living through formal employment may have no choice but to rely on male family members and carry out unpaid care responsibilities without any bargaining power. Secondly, this model implies that women may not be given sufficient opportunities to engage in formal employment. The government may not play an active role in assisting women to become workers through policy measures, such as the provision of childcare services and paid maternity and/or parental leave [2,18].

Nevertheless, even if the adult worker model is implemented, there is no guarantee that this alternative model will fit better with people’s diverse work/family needs or that adults can be freed from all the defects of the traditional male breadwinner model when organising their work and family lives. Some socio-economic conditions are not favourable for the effective implementation of government measures based on the adult worker model. For instance, although evidence shows that paid paternity leave has the potential to encourage fathers to share care responsibilities within the family, many fathers do not take this leave (or do not take the full leave period allowed) due to conventional gender ideologies and societal pressure that expect them to take on the breadwinner role [19]. In other words, despite the fact that the model is presented in gender-neutral terms, existing gendered constraints remain unaddressed by policy measures [5].

Additionally, the adult worker model does not necessarily translate into policy measures that benefit individuals with non-normative family forms. A recent study comparing opposite-sex and same-sex couples’ entitlements to paid parental leave in 34 OECD countries shows that same-sex couples remain disadvantaged when accessing paid parental leave [20]. Without legal recognition of their partnerships, same-sex couples in some countries, such as South Korea and Turkey, cannot enjoy equal paid parental leave benefits, which are available to married couples. Even in jurisdictions that legally recognise same-sex partnerships, differential access to paid parental leave persists due to the insensitivity of policies to different family forms, such as gender-restrictive parental leave policies in a few countries that incentivise leave for fathers without providing equivalent options for same-sex female couples. Moreover, in countries that allocate a longer period of leave to mothers than to fathers, such unequal placement of childcare responsibilities on women also disadvantages same-sex male couples who attempt to access parental leave [20].

Together, these examples highlight the limited applicability of the adult worker model to individuals and families with diverse needs. In particular, this limitation can be attributable to persistent heteronormative and gendered assumptions about family life and care responsibilities, which perpetuate not only gender inequalities between women and men but also inequalities between same-sex and opposite-sex couples. Heteronormativity, as conceptualised by Berlant and Warner [21], refers to ‘the institutions, structures of understanding and practical orientations’ that lead to heterosexuality being privileged and endowed with ‘invisible, tacit, society-founding rightness’ (p. 548).

In the next section, we discuss the heteronormative and gendered constraints confronting lesbians in China. To date, no research has brought the study of the challenges faced by lesbians in their family and work lives into dialogue with discussions of the adult worker model. In order to increase different people’s chances of having sufficient freedom to choose their modes of participating (or not participating) in the work economy as a worker and in the family as an adult, it is necessary to be more cognisant of the challenges faced by different communities.

### 1.2. Gendered and Heteronormative Constraints Faced by Lesbians in China

Previous research has shown that the rapid societal changes in China, particularly the introduction of market-oriented reforms since 1978, have brought new opportunities for women, including lesbians, to develop their potential through increased access to higher education and career advancement [8,22]. Nevertheless, lesbians continue to be in a disadvantaged position as they navigate their family and work lives. Although homosexuality per se has never been criminalised in China, one of the biggest hurdles confronting Chinese lesbians (and gay men) is the pressure to marry the opposite sex imposed by their family of origin [8,23]. Many parents are still strongly influenced by traditional Chinese family-oriented culture, which emphasises that every adult man should have a wife and every woman should have a husband and that an opposite-sex married couple should raise children as a kind of pension for their later life stages [23,24]. In this sense, entering opposite-sex marriage is regarded as more of a social obligation than a personal choice [25]. It is thus not surprising that lesbians who fail to meet this social obligation are judged negatively.

In theory, taking part in the work economy may give lesbians financial resources to lead their own lives and achieve financial independence from their families of origin. However, in practice, it is not easy for lesbians to establish a foothold in the work economy due to discrimination against them. The fear of judgement compels many lesbians to hide their sexual orientation in the workplace [25]. However, this may not necessarily save them from trouble. They are likely to be seen as single women and stigmatised as ‘leftover women’ if they are not yet married by their late 20s [7,8]. In the workplace, lesbians are thus doubly marginalised by the heteronormative discourse of marriage and family due to their non-heterosexual identity and their single status.

Another major challenge confronting lesbians and other sexual minority people is the absence of any legal recognition of same-sex marriage [8]. Same-sex couples are also not legally allowed to use assisted reproductive technology (ART) to have their own child(ren), or to adopt a child. As a result, those who form their own same-sex families are given no place in the welfare system. Some lesbians and other sexual minority people have launched individual and collective actions to demand the legalisation of same-sex marriage [26]. Nevertheless, not only has the government shown no inclination to legally recognise same-sex partnerships, but it has also tightened its control over lesbian, gay, bisexual, and trans (LGBT) movements in recent years [27,28].

Taking the current socio–political context of China and the marginalised position of lesbians into account, this study is intended to answer two research questions: (1) What are the difficulties faced by lesbians when choosing how to take part (or not take part) in the work economy as a worker and in the family as an adult in China? (2) How do lesbians respond to these difficulties? As we demonstrate with our findings, discussing these difficulties and responses provides new insights into possible ways of enhancing the applicability of the adult worker model. It is necessary to expand policy measures that can support the adult worker model and, equally importantly, to create favourable socio–political conditions that not only promote people’s right to work but also recognise same-sex couples’ right to form and care for their own families and enhance their chances of attaining parental and societal recognition.

## 2. Methods

This article draws on data collected through semi-structured interviews conducted in 2017 and social media analysis of articles published on two Chinese online platforms between 2018 and 2020. The qualitative mixed-methods design aims to provide a broader and deeper understanding of lesbians’ work and family lives from both individual and community perspectives.

With the goal of capturing lesbians’ lived experiences in the domestic and work spheres, and their ways of making sense of these experiences, interviews were conducted with 20 lesbian-identified participants in Beijing, the capital of China. Participants were recruited through LGBT organisations which have established rapport with the local lesbian community, and through snowball sampling with the assistance of personal networks and participants’ referrals to their own networks. Participants were aged between 20 and 30. Six of them were local, with household registration in Beijing, while the rest had migrated from other Chinese cities to Beijing for work or study. Six participants had a master’s degree and 12 had an undergraduate degree. At the time of the interview, two participants were studying at university, two were self-employed, and the rest were working in a variety of sectors, namely design, information technology, the media, and NGOs.

Each participant received an information sheet about the study and signed a consent form before being interviewed. The interview guide covered a wide range of topics, including participants’ self-perceptions of their lesbian identity, their ways of handling family relationships, their experiences in the workplace, and their views of government policies and the social climate. Participants were able to respond to the interview questions at their own pace and freely express their ideas beyond the interview guide. All the interviews were audio-recorded and then transcribed. Pseudonyms were used to ensure confidentiality.

In addition, we collected and analysed social media data on two major lesbian-related WeChat platforms, ‘the Beijing LGBT Centre’ and ‘Rainbow Babies’. WeChat is the most popular instant communication tool and one of the most widely-used social media platforms in China [29]. The aim of the social media analysis was to identify a wider range of work-related and family-related topics discussed by the online lesbian community. Notably, the interview data has limitations because it primarily covers the experiences of a sub-group of lesbians living in Beijing, who were willing to share their experiences with the researcher face-to-face. A growing number of qualitative scholars are suggesting that social media data are valuable as a new source of data that can complement traditional qualitative approaches [30]. Such data can add new dimensions to interview data because online platforms can reach participants from across a wide geographical area, particularly hard-to-reach populations, and, more importantly, they offer an open environment for participants to express deeply held personal opinions and discuss sensitive issues anonymously [31]. Capitalising on these strengths, our study analysed WeChat online articles with the aim of gaining deeper insights into the social issues and/or policies that concern a wider range of lesbians and the views held by lesbian community leaders. Moreover, as the majority of our interview participants, who were in their 20s, were exploring intimate relationships and none of them reported pursuing motherhood at the time of the interview, we turned to the social media data to further investigate lesbians’ fertility and family-building plans, which remain largely silenced in Chinese society and the existing literature [27].

The two WeChat platforms were selected because the Beijing LGBT Centre is the largest LGBT organisation in China and Rainbow Babies is the best-known platform for lesbians who plan to have children and those with children. It is noteworthy that the ethics of social media research remains a contested territory [32]. While we were aware of other LGBT-related WeChat groups, where communication about different LGBT family-related topics took place, we deliberately did not include them because these discussions were of only a semi-public nature in the sense that access needed to be granted by the group owners. By contrast, the two selected WeChat platforms were publicly visible and accessible. We created pseudonyms for the (already pseudonymised) articles presented here. All the articles on the WeChat platforms were regarded as authored, culturally produced texts [33], which enabled access to the norms and community practices concerning how to live as a sexual minority woman in China.

Between 2018 and 2020, there was a total of 1803 articles published by the Beijing LGBT Centre and 82 by Rainbow Babies. For data analysis, our research team selected 197 articles published by the Beijing LGBT Centre and 60 by Rainbow Babies. They were selected for their relevance to the economic and intimate aspects of lesbians’ lives, such as articles pertaining to employment discrimination, same-sex marriage, and reproduction. This focus was guided in the first instance by our research questions and then by the findings of the interviews, which shed light on lesbians’ interconnected experiences in the domestic and work spheres.

The findings presented below were drawn from the main themes developed from the interview and social media data. Based on Braun and Clarke’s [34] guidelines, thematic analysis was adopted to generate codes and themes from this data. We analysed the responses of participants in the interviews, identified the commonalities and differences between these individual responses, and subsequently compared and contrasted them with the contents of the articles published online by the two social media platforms. To enhance the credibility of the data analysis, all the researchers actively reflected upon and questioned their assumptions by comparing the two sources of data and discussing their interpretations. They also collected feedback on the findings from some of the interview participants in order to reflect upon their analysis. We chose the specific cases discussed here because they best illustrate key aspects of the themes. 

## 3. Findings

### 3.1. The Perceived Importance of Wealth and Status in Earning Recognition and Defying the Male Breadwinner Model

Our participants’ narratives, coupled with the social media data, illustrate three major reasons for lesbians to join the labour market. Firstly, lesbians tend to rely on financial resources to enhance their social and spatial mobilities within and across cities, taking parental control and gender norms in Chinese society into consideration. Secondly, they are inclined to work particularly hard in order to counter the stigma attached to homosexuality and to earn parental and societal recognition as capable adults in the labour market. Thirdly, lesbians may turn to the market to resolve their practical needs, such as fertility and migration plans, in order to support themselves in leading their preferred way of life.

For instance, during her interview, Zoe (aged 23) stressed that she would have to surrender control over her own life if she still relied on financial support from her parents. With a career in the Internet industry, Zoe reported working long hours every day with the goal of gaining recognition from others. Her job was also crucial to her because it had enabled her to afford to rent a flat with her partner and move out of her parents’ home in Beijing. The strong desire to escape from parental control was widespread among participants. Consistent with previous studies, Chinese women continue to be subject to traditional gendered norms imposed by parents, who expect their adult daughters to live with them until they enter an opposite-sex marriage [8]. This traditional gendered expectation can also largely explain why all the non-local participants reported moving to Beijing to distance themselves from their families of origin and enjoy greater freedom.

Participants’ determination to thrive in the labour market is also in line with previous studies, which show that Chinese sexual minority people tend to see financial success as a precondition for coming out as respectable [8,25,35]. The vast majority of our participants had not yet come out to their parents, but accumulating enough money was commonly considered a prerequisite for earning familial recognition. Zoe explained that financial capital could enable a lesbian to be treated as an independent and capable individual within her family of origin and thus to come out with a higher chance of being accepted:

*‘With a sufficient amount of money, I can earn people’s recognition that I am a person of great ability… If I come out to my parents later, there will be a greater chance of earning their acceptance.’*

Another participant, Cara (aged 23), who was a student, also believed that having a successful career was indispensable for gaining people’s respect for her sexual identity, which remains heavily stigmatised in Chinese society. With ‘money and power’, she believed that she would then have less trouble coming out to her parents. She said: ‘Only those rich and powerful ones can come out and hold their heads high. We need to work extra hard to achieve that goal’.

The discourse of the importance of financial independence and affluence also permeates discussions about intimate and family life on the online platforms of Rainbow Babies and the Beijing LGBT Centre. It is noteworthy that none of the research participants were mothers at the time of the interview, largely because same-sex couples are denied the right to ART or adoption by law in China [8]. Meanwhile, the social media data enabled us to gain access to the experiences of Chinese lesbian mothers, who remain a hidden and hard-to-reach population. For instance, in an online article entitled ‘This is a Baby who Can Save Money’, the founder of Rainbow Babies, who is a mother raising two children with her same-sex spouse (they married overseas), shares her personal experience of seeking ART overseas and her financial advice on how to choose ART clinics and compare the costs, quality, and effectiveness of ART services in different countries. The title and content of this online article emphasise the key role of financial resources in lesbians’ fertility and family plans. While same-sex intimacy and parenthood have long been considered mutually exclusive in traditional Chinese society [27], the online articles published by Rainbow Babies show that accumulating financial resources and becoming an informed consumer in the global ART market enables some lesbians to become both breadwinners and parents. Nevertheless, given that access to assisted reproductive options is segregated, particularly along lines of class and cultural capital [36,37], only a small proportion of lesbian adult workers are likely to have the opportunity to realise their reproductive and family plans.

Another important insight is that, compared with the interviews, the social media data offers additional insights into a wider array of experiences associated with the meaning of work and the role of wealth and power in one’s coming-out plan. Written with a community perspective, one of the heated topics in online articles published by the Beijing LGBT Centre is the coming-out journeys experienced by lesbians (and gay men). On the one hand, similar to the view shared by the majority of participants such as Cara and Zoe, these articles show that making a decent living plays a key role in generating symbolic power to convince parents of the positive prospects of a non-heterosexual intimate relationship. On the other hand, these articles are often written with a note of caution—whether and how to come out varies between individuals and should depend on individual circumstances. This needs to be understood within the context of the Centre’s ongoing work in handling cases of lesbians being rejected by their parents, and even having parents’ financial support terminated after coming out, which was reported by one of the participants during her interview. The following excerpt from an article posted by the Beijing LGBT Centre presents an example of how lesbians make sense of their work lives in relation to the need to come out in different ways. Yu, a lesbian volunteer at the Centre, reported that she was unwilling to come out to her parents due to fear of disappointing them, but she was determined to build a family with her same-sex partner. In this article, she shared the ‘success’ story of her lesbian friends:

*‘Both of them are now 37 years old and have moved to Canada. Although they have never come out [to their parents], they are strong enough to live a decent life, and at the same time, let their parents know about their living conditions and the fact that this “friend” is really reliable. I see this as probably the best compromise, the best solution to achieving a balance between parents and themselves.’*

Rather than directly coming out, Yu believed that, by earning enough money in the labour market, lesbians could demonstrate that they were ‘reliable’ adults who were capable of taking care of themselves and their partners. Their ability to be self-sufficient and defy the male breadwinner model might convince their parents to acquiesce to, if not formally recognise, their intimate relationships. This echoes previous findings that the liberal ‘out and proud’ model featured in Western literature has its limitations when seeking to understand how Chinese people perceive, disclose, and react to non-heterosexual identities [8,38]. Studies have shown that openly disclosing one’s sexual identity is deemed too confrontational by some Chinese lesbians (and gay men) [8]. To lesbians, earning money can be considered a means to an end—an end not necessarily associated with verbally coming out as a sexual minority woman, but one that enables a lesbian to be recognised as an independent adult who can decide upon her own family life. It can potentially help lesbians to resist persistent patriarchal and heteronormative expectations, which confine women to the roles of dutiful wife and family caregiver under the traditional male breadwinner model [8].

Furthermore, while our interviews focus on lesbians residing in Beijing, almost all of whom were devoted to developing their careers in China, the social media data point us towards other motivations behind hard work in the labour market—the prospect of moving to Western countries, where same-sex marriage is legally recognised. Recent studies have begun to explore how sexuality and class might influence migration experiences [22]. For instance, focusing on queer Chinese women, including lesbian, bisexual, transgender, and queer-identified women, who migrated from China to Australia, Kam [22] discussed how their experiences of migration were anchored to ‘the neoliberal desire to be a mobile cosmopolitan subject’ (p. 126). The fact that Yu and her friends wanted to migrate to a Western country and that there are many online articles published by the Beijing LGBT Centre about the legalisation of same-sex marriage in other countries demonstrates that lesbians (and gay men) may resort to the market to achieve more freedom through migration and to circumvent parental and societal disapproval of homosexuality. Nevertheless, this ‘new class of mobile urbanites’, as Kam suggests [22] (p. 131), is confined to a group of lesbians within the middle or upper class, who are capable of planning a life abroad. It excludes many of the lesbians who have encountered gendered and sexual obstacles in the labour market, to whom we now turn.

### 3.2. Vigilance about Gendered and Heteronormative Expectations in the Workplace and the Need for Structural Change

It was common for participants to express constant vigilance about the heteronormative environment in the workplace and the gendered and sexual biases displayed by their colleagues. Most participants shared the belief that, until they had achieved a higher social status and were powerful enough to have a greater say, they had no choice but to follow the heteronormative expectations of those people with power and status.

Having worked for a state-owned enterprise for a few years, Hailey (aged 28) reported feeling uncomfortable in the workplace and reluctant to come out to her colleagues due to fear of risking her career prospects. She reported hearing hostile gossip and judgements about non-normative gender expressions and sexual identities in the workplace. She raised an example of unfair treatment faced by a former colleague, Vicky, who was open about her lesbian identity and had a masculine gender style. Based on Hailey’s observations, her colleagues often asked Vicky to work overtime and do manual labour, such as moving furniture and packing things. Hailey believed that this unfair allocation of tasks was related to Vicky’s masculinity and sexual identity, which fell outside the norm. While such unfair treatment was not targeted at Hailey, it was evident that she was vigilant about the fact that any departure from heteronormativity might have an impact on how she was treated. The need to constantly scan for signs of trouble or threats eventually prompted her to leave the state-owned enterprise and find another job at an LGBT-friendly company.

Participants shared the view that lesbians with a masculine gender style tended to face more unfair treatment than lesbians with a feminine gender style. Some participants, such as Zoe (aged 23), Betty (aged 26), and Ariel (aged 30), embraced a masculine gender style by having short hair, often wearing men’s clothes, and not wearing makeup, dresses, or high-heeled shoes. Since they did not follow the stereotypical expectations of femininity, they were challenged by family members and colleagues for choosing not to dress themselves up in a feminine way. Non-conforming gender expressions pushed them into a disadvantaged position in the workplace.

Moreover, lesbians are vulnerable to discrimination in the workplace due not only to their sexual identities and gender expressions but also to their unmarried status. Working for an international public relations company in Beijing, Vivian (aged 24) stressed that she had never come out to any of her colleagues in order to avoid unfair treatment. She said: ‘She [the boss] might ask me to take up rotten tasks and treat me poorly. You never know. It’s better to hide my sexual orientation’. She also reported being asked about her marital status during her job interview. This shows that one’s personal life is evaluated as part of the work relations in the heteronormative labour market. She also added that married employees were often assumed to have care responsibilities in their families, and consequently unmarried employees tended to be asked to work longer hours and carry more of the workload. She said: ‘If we could disclose our sexual orientation at work without worrying, we would not need to put up with this’. Such unfair allocation of tasks, in turn, reduced her free time and opportunities to develop intimate relationships outside the workplace. Moreover, consistent with the existing literature revealing the common practice of matchmaking among heterosexual people by means of introductions through not only parents’ networks but also those of co-workers in China [39], some participants had no choice but to attend matchmaking dates with men introduced by their supervisors in order to please their supervisors and avoid arousing suspicion about their sexual orientation. In short, participants were expected to display femininity and perform heterosexuality in order to develop good work relations in the labour market.

A few participants believed that they could exercise their choice to work for an LGBT-friendly company (such as Hailey), or to set up their own businesses, through which they could fit in with the neoliberal environment as self-enterprising subjects [40]. Such agency, however, needs to be understood within the specific context of the neoliberal market economy in China, where people are expected to both obey authoritarian rule and become self-enterprising subjects in pursuit of wealth and personal advancement [40,41]. In other words, instead of questioning the lack of legislation protecting sexual minority people from discrimination in China, lesbians (and gay men) tend to accommodate heteronormative expectations and navigate their own routes for surviving the competitive market. As shown by the interview data, having experienced or witnessed unequal treatment based on one’s gender expression, sexual orientation, and/or marital status in the workplace led participants to cling to the belief that wealth and status through their ‘choices’ of work would be the best weapons to fight oppression and the key to maintaining the intimate and family relationships they desired as lesbians.

While the interview data reveal participants’ experiences in the workplace and their ways of making sense of and responding to heteronormative expectations, the social media data provide an additional perspective on how the sexual minority community may attempt to resist heteronormativity. Discourses centring upon collective efforts and hope for change abound in the articles published by both the Beijing LGBT Centre and Rainbow Babies. On the one hand, the Beijing LGBT Centre shares the findings of external reports about the experiences of discrimination faced by LGBT people in the workplace. For instance, one of the online articles shares data published by the United Nations Development Programme China Office, and alerts readers to the findings that over 60 percent of LGBT job applicants reported being excluded from the application process and about 10 percent attributed their application failure to their gender identity and/or sexual orientation. On the other hand, the Beijing LGBT Centre also calls attention to the possibility of challenging the status quo. An article entitled ‘I came out in front of over 200 people: How to come out in the workplace’, published by the Centre, features Rui, who shared her experience of working for an LGBT-friendly multinational corporation with over 200 university students at the corporation’s recruitment event. The fact that there were LGBT-supportive policies within the corporation, such as clear guidelines on how to protect employees from discrimination on grounds of sexual orientation, made Rui feel that she was supported and valued as a lesbian employee. She described how the LGBT-friendly climate had changed her views on intimate and family life:

*‘In the past, I never dared to think about the future with my partner, getting married, or starting a family, which seemed so far out of reach. Coming out [in the workplace] has brought about a chain reaction. I no longer fear and avoid intimate relationships as I did before. It made me see a lot more possibilities. It made me more optimistic and more courageous about imagining the future.’*

Rui came out to her parents after coming out in the workplace because she believed that she was able to prove to them that her sexual identity would in no way jeopardise her career. This article demonstrates how individual experience as an adult worker, especially that of coming out and being accepted in the workplace, may have a profound impact on other aspects of life as well, such as intimate and family relationships. It also highlights the importance of structural support in the workplace. Another article on workplace experiences published by the Beijing LGBT Centre echoes this point: ‘It is not that you are not working hard enough; In fact, when there is structural discrimination, no one is able to “work hard” to fight it.’ Without an inclusive workplace climate and policies, many lesbians continue to be marginalised in the labour market due to their single status, non-normative sexual orientation, and/or gender expression.

### 3.3. Challenges as an Adult Worker and a Parent: The Lack of Welfare and Social Support

People cannot always support themselves through participation in the labour market. Due to various reasons, such as illness, old age, or pregnancy, they need to find ways to maintain a reasonable standard of living independently of labour force participation [42,43]. This implies that institutions such as the family and the government should play a significant role in supporting individuals. However, due to their non-normative sexual orientation, it is difficult for lesbians to seek help from either their families of origin or the government. Moreover, given the fact that existing family scripts are still centred around the heterosexual family model, participants found it difficult to imagine themselves having a family life, and some of them reported never thinking about the prospect of becoming a parent. The belief that earning money, rather than having a child, was the ‘top priority’ was commonly shared by the participants during their interviews.

The social media data enable a more thorough examination of the barriers to parenthood faced by working lesbians. Given the lack of legal status of lesbian couples (and their children) as a family unit and the absence of societal and state support for lesbian mothers, lesbians who can afford to use ART overseas tend to face a dilemma, in which they have no choice but to sacrifice their career for their family life if they decide to have children. For instance, in an article on the Rainbow Babies platform, a lesbian mother shares her intense struggle to give up her career in order to take up caring responsibilities for her child, who was conceived through ART overseas and raised by herself and her partner, who remains in her job:

*‘Giving birth to a child and raising him/her will probably take me three years or even longer, during which I can’t work. My career, which I’ve been working hard at for ten years, will just vanish. What can I do? Until the moment I resigned, I hadn’t completely thought it through; or, let me put it this way, I didn’t have the courage to face the life of a full-time housewife with neither a source of income nor a career.’*

This remark is typical of discussions among intended lesbian parents who share the same dilemma: at least one member of the lesbian couple has to quit her job to take up full-time caring duties for the prospective child(ren). On the Rainbow Babies platform, the ‘worries’ and ‘anxiety’ felt by lesbians about their future re-engagement in the labour market and career development after being full-time family carers for several years are common topics. These difficulties can be attributed to the heteronormative work environment and a welfare system that does not recognise a lesbian couple (and their children) as a family unit. Whereas heterosexual mothers are entitled to maternity leave and their male spouses are entitled to paternity leave by law in China, lesbians who are pregnant struggle to claim maternity leave benefits due to heterosexual norms in the workplace. As shown in the previous theme and existing research [25], lesbians in China tend to hide their sexual orientation in the workplace due to perceived and actual discrimination. Worse still, it is particularly difficult for lesbian mothers who have remained in the closet and are thus perceived as single women, to explain to their co-workers how they have become mothers and to claim maternity leave. It is also impossible for prospective non-biological lesbian mothers to apply for parental leave.

Despite these difficulties in reconciling work and family due to the lack of welfare and social support, the Beijing LGBT Centre suggests that a growing number of lesbians have the desire to form their own families with children. Meanwhile, the Centre illustrates the financial and emotional burden borne by lesbian couples along their paths to parenthood. Not only do they need to go to extraordinary lengths to seek ART overseas, which is expensive and may derail their careers, but they also need to face the lack of legal recognition of the familial and parental status of same-sex parents. While biological lesbian mothers can prove their genetic ties with their children, non-biological mothers do not have the opportunity to enjoy equal status as parents in China. For instance, one of the Beijing LGBT Centre’s online articles features the experience of a non-biological lesbian mother, whose child was conceived by her partner through ART, and her view on legal protection:

*‘We believe that we build parent-child bonding through everyday interactions. But undeniably, the lack of legal recognition of our relationship makes me feel unsure of myself when I face challenges. If our family could be legally recognised, I wouldn’t feel stressed at all, despite the lack of blood ties. We really need to work hard and go out of our way to live a happy life, just as ordinary people do.’*

By sharing stories about lesbians’ difficult experiences of becoming parents, both the Beijing LGBT Centre and Rainbow Babies platforms call for more attention to be paid to the needs and wants of lesbian (intended) parents and advocate policy change to address unequal reproductive rights. Notably, gaining access to ART overseas requires economic privilege and access to resources in order to become a consumer in the transnational intimate economy of assisted reproduction. It is almost impossible for lesbians to seek financial support from their parents to purchase ART due to the deep-rooted belief that only opposite-sex marriage is the gateway to parenthood [44,45]. In other words, the option of motherhood is less imaginable, or even unimaginable, for poorer lesbians, who may struggle to fulfil gendered and heteronormative expectations and earn a decent living through the labour market and, consequently, fail to become beneficiaries of the neoliberal ideal of empowered consumerism.

Written by the founder of Rainbow Babies, an online article entitled ‘As Women, As We were Born Poor, As We Love the Same Sex’ illustrates the popular ‘myth’ of the power of wealth and status to bring about ‘liberation’: ‘We are more inclined to believe that financial freedom will liberate us. Compared with those in the majority, we seem to be more convinced of the myth of external labels that are attached to wealth and status’. As shown in this sub-section, lesbians who want to become mothers have no alternative but to empower themselves through the market, including (re-)entering the competitive labour market and becoming consuming subjects in the global ART market. Meanwhile, even though some lesbians can afford ART, prospective lesbian mothers are likely to be pushed into a disadvantaged position in the workplace compared with their heterosexual counterparts.

## 4. Discussion

This research provides fresh insights into the difficulties faced by lesbians in their work and family lives in China. Based on the interview and social media data that we gathered, the disadvantaged position of lesbians as both women and sexual minority individuals greatly reduces their chances of thriving as self-sufficient workers in the labour market, being recognised as independent adults by their families of origin, and receiving welfare and legal support as parents. Nevertheless, the challenges faced by sexual minority women are missing from the discussion of the adult worker model, which has primarily been developed on the basis of heteronormative assumptions. Below, drawing on the empirical evidence, we discuss the limitations of the applicability of the adult worker model.

### 4.1. Challenging the Male Breadwinner Model: Difficulties in Being Recognised as an Independent Adult and Becoming a Self-Sufficient Worker

While China’s entry into the global neoliberal market has encouraged more people, including those in the LGBT community, to aspire to become self-enterprising agents and achieve upward mobility through hard work [41], Chinese lesbians continue to encounter not only gender-based injustice but also other types of injustice due to their non-normative sexual orientation. These injustices adversely affect lesbians’ chances of becoming ‘adult workers’ who can be recognised by their families of origin and respected in the neoliberal market. As shown in the findings, lesbians generally experience great parental and societal pressure to marry the opposite sex in order to become qualified as ‘proper’ adults. There is still intense control over adult children, and especially over women, in Chinese families [8]. As a result, it is not uncommon for Chinese lesbians to express a strong desire to earn their own living, move away from their hometowns, and even migrate to other countries in order to enjoy more personal space and pursue the adult life they want [8,22]. Their inclination to work particularly hard in the workplace also echoes previous Western research, which shows that lesbians tend to show more dedication to the labour market than heterosexual women because they are less likely to have a higher-earning partner who could take up the role of sole breadwinner [46,47].

Although lesbians may resort to individual strategies to prove that they are independent adults and defy the male breadwinner model, our findings reveal that lesbians tend to confront predicaments in the labour market in three ways. Firstly, lesbians as women are still expected to strictly follow gendered and heteronormative expectations in the workplace. They are adversely influenced by their unmarried status, such as being questioned about their single status during job interviews and forced to carry an extra workload. Single women are often stigmatised as ‘abnormal’, whereas their male counterparts are much less affected by their single status [48]. Secondly, as suggested by some participants, concealing their sexual identity in the workplace had adverse consequences for their intimate relationships. These included being forced to attend heterosexual dates with men introduced by the boss and to work for longer hours due to their perceived single status. They hid their sexual identity to avoid jeopardising their job and promotion opportunities, but at the same time, their opportunities to find a potential partner or maintain a same-sex relationship were hampered by this devotion to their careers. Thirdly, lesbians who want to be, or have become, mothers within same-sex relationships are denied access to parental rights, including paid leave. At least one parent within the couple may have no choice but to sacrifice her career for motherhood due to the necessity of meeting all care responsibilities without welfare support. It is also noteworthy that only those who possess sufficient material and social resources may have the option to seek ART overseas and become parents [27]. As shown in previous studies, neoliberal rhetoric, which centres upon aspirations to be respectable adults and achieve upward mobility, may continue to reproduce hierarchical divisions within the lesbian (and gay) communities along the lines of class, the rural–urban divide, and other social divisions, without challenging heteronormative values [22,49].

### 4.2. Implications for the Adult Worker Model

Our findings show how gender norms, heteronormativity, and government policy intersect in generating obstacles for Chinese lesbians to thrive as respectable adult workers. It is, therefore, important to implement structural changes within and beyond the workplace in order to address gendered and heteronormative injustices. In this regard, it is necessary to consider expanding the adult worker model. Firstly, measures to deter and punish discrimination against women on the grounds of marital and family status are urgently needed in the workplace. Moreover, as shown in our data, promoting LGBT-supportive policies in the workplace can make lesbian employees feel valued and supported and rid them of the fear of coming out to colleagues and family members. Ensuring equitable access to employment and promotion opportunities for lesbians (and other sexual minority people) serves to create more favourable socio-economic conditions for the development of the adult worker model. It is important not only to provide legal protection for sexual minority people against discrimination but also to grant them the right to legal partnership. Furthermore, to enhance the applicability of the adult worker model to individuals and families with diverse needs, policymakers should be sensitive to heteronormative and gendered assumptions about family life and care responsibilities. For instance, when drafting family-related policy measures, it is necessary to use gender-neutral language that does not assume women to be family caregivers and to extend parental leave entitlements to all parents in different forms of families [20].

Although China falls short of the welfare support provided by advanced welfare states in some European countries and lacks social welfare for sexual minority people, our research has important implications for the development of an inclusive and democratic form of the adult worker model. Such democratisation of the model requires collective efforts to remove the gendered, heteronormative, and other institutional barriers that hinder sexual minority women from exercising choice over their work and family lives. This article suggests that social policy that is mainly driven by goals for economic development is too one-dimensional and thus may lose sight of the welfare of citizens, especially that of socially marginalised groups. Although the emphasis of the market-focused adult worker model is placed on labour force participation and the economy, it has overlooked the additional difficulties and complex social dynamics faced by sexual minority women when they engage in paid work, as evidenced by our findings. While it is necessary to encourage women to exercise their own choice in determining their work and family lives, it is equally important to ensure that women, especially sexual minority women, are able to make those choices within a discrimination-free work and social environment.

Further research is needed to study the tension between individualisation and familisation, especially when it comes to the formation of families. As shown in our research, lesbians’ complex entanglement of familial and economic lives demonstrates the oversimplification of the adult worker model, which assumes women to be individualised market subjects and thus immune to the traditional expectations prescribed by the male breadwinner model. Additionally, scholars have recognised that there is a gap between the adult worker model and the complex realities of family lives [5]. Despite the erosion of the old male breadwinner model, it is still rare to find families comprised of fully individualised and self-sufficient members, each of whom engage in the labour market on a full-time basis and are independent of one another. There is still asymmetry in men’s and women’s roles within the family, with women carrying out more unpaid care work than men [50,51]. In other words, the treatment of women as independent workers as proposed by the adult worker model is an oversimplified view of family relations that ignores the normative expectations imposed on women as well as their own sense of commitment to their families, which may restrict their autonomy to make their own decisions [12]. While this study only focuses on Chinese lesbians, future studies can build on it to examine the work and family lives of other groups who live outside the conventional heterosexual family, such as those of single mothers and gay men, in different cultural contexts and welfare regimes.

## 5. Conclusions

In bringing the empirical evidence from this research into dialogue with the literature on the adult worker model, we have discussed the limitations of the model and ways of enhancing its applicability to women and families outside the heterosexual family framework. This article highlights the gender-based and sexual-orientation-based obstacles faced by lesbians and their ways of responding to the difficulties of simultaneously taking part in the family as an adult and in the work economy as a worker in China. Couched in gender-neutral terms, the adult worker model has largely overlooked the interplay of gender and sexuality factors in shaping work and family lives. The findings shed light on the discussion of the adult worker model, providing a new lens through which to explore the interaction between the state, the market, and intimate and family relationships and to identify directions for creating more inclusive, alternative models.

## Data Availability

Data are contained within the article. The whole dataset is not publicly available due to ethical considerations.
